# Occurrence of anticancer drugs and widely used pharmaceuticals in sewage sludge, compost, and river sediment

**DOI:** 10.1007/s11356-026-37898-3

**Published:** 2026-06-04

**Authors:** Olga-Sofia Alitalo, Ekaterina Gutovskaia, Anna-Lea Rantalainen, Jukka Pellinen

**Affiliations:** Department of Environmental Sciences, Faculty of Biological and Environmental Sciences, University of Helsinki, Niemenkatu 73, Lahti, 15140 Finland

**Keywords:** Anticancer drugs, Emerging contaminants, Pharmaceuticals, Sewage sludge, Wastewater treatment, Compost

## Abstract

Pharmaceuticals enter the environment mainly via municipal wastewater treatment plants, where some compounds bind to sewage sludge during treatment processes. The contaminated sludge often undergoes fermentation and composting and is later used as a soil amendment. This study aims to investigate the fate of five pharmaceuticals letrozole, tamoxifen, carbamazepine, diclofenac, and naproxen, in environmental solid matrices, including sewage sludge, compost, and sediment. An analytical method based on microwave-assisted extraction, SPE, and UPLC-MS/MS was developed and validated. The most abundant compounds in sewage sludge were carbamazepine (29–92 ng/g dw) and diclofenac (46–213 ng/g dw). Tamoxifen occurred at lower concentrations (5–19 ng/g dw), while letrozole and naproxen were generally detected below LOQ levels. Carbamazepine and diclofenac were the only compounds detected in compost samples. In the receiving river sediment, studied compounds were found at low concentrations (< 11 ng/g dw), except letrozole and tamoxifen, which were not detected. The results suggest that carbamazepine and diclofenac bind to sewage sludge and are not degraded during treatment processes, potentially ending up in compost and soils. This emphasizes the need for further research on their environmental impacts at low concentrations in soil.

## Introduction

Pharmaceuticals belong to the group of emerging contaminants (ECs) which potentially pose a threat to the ecology of receiving environment. Pharmaceuticals are used for both human and veterinary healthcare and their consumption is likely to increase in future years with an aging population and improved quality of life worldwide (Van der Aa and Kommer 2010). Although the environmental fate of pharmaceuticals has been widely studied during the last decades, their significance has been highlighted more recently due to advancements in analytical techniques, which have allowed for their detection and quantification at extremely low concentrations. Consequently, pharmaceuticals have been found in numerous surface water bodies, groundwater, and terrestrial environments globally provoking special concern due to lack of regulation on the discharge of these persistent and widespread chemicals (Yang et al. 2011; Topp et al. 2008; Corada-Fernández et al. 2015; Almeida et al. 2021). Despite the ongoing research, the potential environmental impacts of many pharmaceuticals remain poorly understood.

Pharmaceuticals enter the environment through various pathways, but wastewater treatment plants (WWTPs) play a significant role. During wastewater treatment processes, pharmaceuticals can mainly be affected by three mechanisms: volatilization, biodegradation, or sorption onto sludge, depending on the physicochemical properties of the compound and the sludge (Seira et al. 2013). Volatilization of pharmaceuticals is usually not expected, while biodegradation and sorption to sludge or remaining in the dissolved form in aqueous phase are the main pathways for pharmaceuticals in WWTPs (Clara et al. 2005; Seira et al. 2013; Mohapatra et al. 2014). Sewage sludge is generated in primary and secondary clarification and in biological treatment processes, like activated sludge process. Typical treatment of sewage sludge involves prethickening, anaerobic digestion—a process where biogas is produced, and dewatering before further processing, like composting. Monitoring the solid phase at WWTPs is important, as sewage sludge contains not only pharmaceuticals but a complex mixture of organic and inorganic compounds, metals, and pathogens (Riffat and Husnain 2022; Yang et al. 2016) and the treated sewage sludge is commonly used as soil amendment, as well as for fertilizers (Malmborg and Magnér 2015; Seira et al. 2013). Composting is a common practice for treating sewage sludge to produce stabilized organic matter suitable for soil amendment. The usage of sewage sludge in agriculture is regulated by European Council Directive 86/278/EEC, and it is complemented by national legislation. In Finland, fertilizer products may contain treated sewage sludge (e.g., composted sludge); however, their use is restricted to the cultivation of plants that are not typically consumed fresh by humans, harvested for their underground parts, or used to feed animals. Therefore, compost that contains sewage sludge is often used for soil improvement in landscaping. Before composting, sewage sludge is often combined with biowaste from households, restaurants, or food industry, and bulking agents are added to increase oxygen flow.

During composting, organic matter in sewage sludge undergoes decomposition by microorganisms under controlled conditions of temperature, moisture, and aeration. Usually, the early stage of composting takes place inside the facilities, in closed tunnels or drum units, and the maturing of compost happens outside for several months. As compost can contain harmful pathogens, compost undergoes hygienization before the maturing phase. The fate of pharmaceuticals in composting depends on factors such as their chemical properties, microbial activity, and composting conditions.

The pharmaceuticals chosen in this study are produced and consumed in large quantities in every part of the globe. Some of the compounds, like nonsteroidal anti-inflammatory drugs (NSAIDs), have been found in various phases of the environment before (Ferrari et al. 2003; Wojcieszyńska and Guzik 2020; Vieno and Sillanpää 2014; Samaras et al. 2013; Isidori et al. 2005). However, some of the selected pharmaceuticals, letrozole and tamoxifen, are seldomly included in scientific studies, even though their usage is considerable, and due to their mode of action and potential carcinogenicity, they may have adverse effects in the environment (Liao et al. 2014; Sun et al. 2007a, 2007b). The selected five pharmaceuticals were hormone antagonists letrozole (LTZ) and tamoxifen (TAM), antiepileptic drug carbamazepine (CBZ), and commonly used nonsteroidal anti-inflammatory drugs (NSAIDs) diclofenac (DCF) and naproxen (NPX). The physicochemical characteristics of these compounds are presented in Table 1. Carbamazepine and diclofenac are of particular interest, as they are included on the European Union (EU) Watch List under the Water Framework Directive (WFD) and are therefore monitored in surface waters across the entire Union.

**Table 1:**
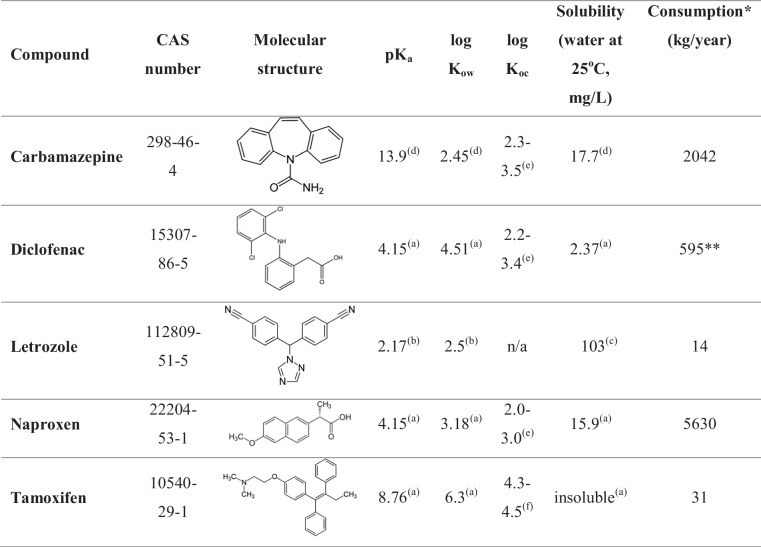
Description of investigated compounds (CAS numbers, molecular structures, pK_a_ constants, octanol–water logarithmic portioning coefficients log *K*_ow_, log *K*_oc_, and solubility

*n/a* not available

^(a)^National Library of Medicine (n.d.)

^(b)^ Liu et al. (2010)

^(c)^ Li et al. (2021)

^(d)^ Vieno et al. (2006)

^(e)^Carballa et al. (2008b)

^(f)^ Yang et al. (2011)

^*^Fimea (2024)

^**^Diclofenac sold as topical gel forms excluded

Understanding the fate of pharmaceuticals in sewage sludge treatment processes is essential for evaluating their environmental behavior and potential risks to aquatic and terrestrial organisms. The aim of this study was to assess the fate of five selected pharmaceuticals in different solid samples from wastewater treatment and receiving river. These include sewage sludge from different processing stages from two WWTPs, sewage sludge as a component of the compost at three different maturation stages, and river and retention pond sediments receiving WWTP effluent. To achieve the aim of this study, an effective extraction method for the quantification of these compounds in various solid matrices was developed and validated.

## Materials and methods

### Chemicals and materials

Standards letrozole, tamoxifen-[D_5_], letrozole-[D_4_], carbamazepine-[D_10_], naproxen-[D_3_], and diclofenac-[^13^C_6_] were purchased from Toronto Research Chemicals (North York, Canada). Tamoxifen, carbamazepine, naproxen, and diclofenac were purchased from Sigma-Aldrich (Steinheim, Germany). UPLC-grade methanol was purchased from Sigma-Aldrich (Darmstadt, Germany) and Honeywell Riedel-de Haën (Germany). Formic acid (HPLC grade) was purchased from VWR Chemicals (Fontenay-sous-Bois, France). Solid-phase extraction (SPE) cartridges Oasis HLB (60 μm, 500 mg, 6 mL) were obtained from Waters (Eschborn, Germany).

### Solid sample collection

**Fig. 1:**
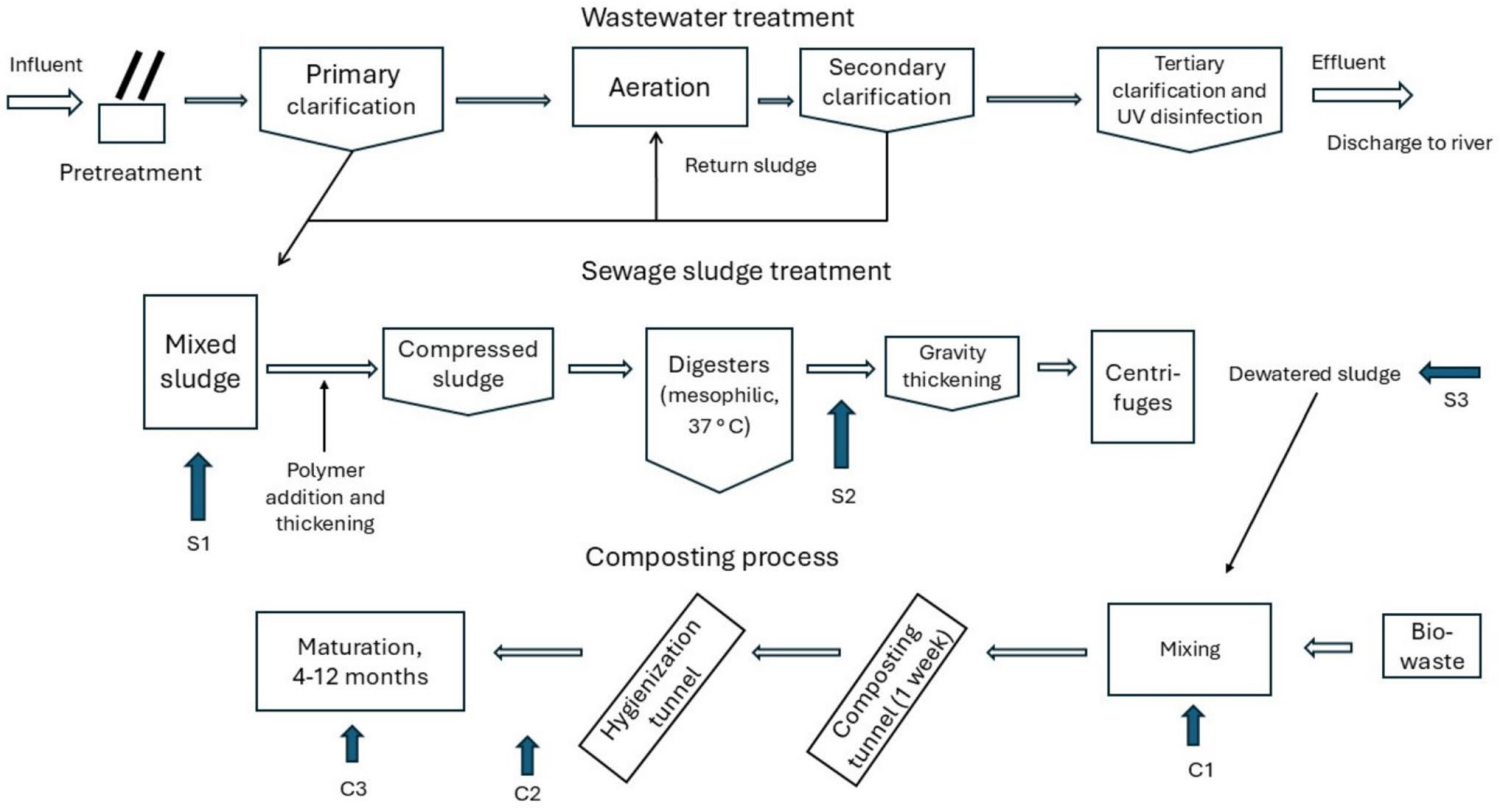
Illustrative process flowchart of wastewater and sludge treatment, following composting. Sampling points for mixed (S1), fermented (S2), and dewatered (S3) sewage sludge samples. Compost samples collected at three different age stages: C1 (0 days), C2 (after 5 days of maturing), and C3 (after 4 months of maturing)

Sewage sludge samples were collected from two local wastewater treatment plants, labeled as WWTP I and II. WWTP I receives wastewaters from households but also from a local central hospital and industry (approx. number of inhabitants 65,000, flow rate 14,936 m^3^/d). WWTP II receives wastewater mostly from households (approx. number of inhabitants 64,000, flow rate 17,845 m^3^/d). Treated wastewater from both WWTPs is directed to a shared retention pond (50,000 m^3^) located near WWTP I; after which, it undergoes tertiary UV disinfection prior to discharge into the receiving river. The disinfection is carried out using a Duron open-channel UV system, and the UV fluence was automatically regulated in real time using the OptiDose control system. Sewage sludge samples from WWTP II were collected in February 2024 and from WWTP I in July 2024. Sewage sludge was sampled in a 5-L plastic bucket from the stages before fermentation (mixed primary and secondary sludge, S1), after fermentation (fermented sludge, S2), and after the dewatering process (dewatered sludge, S3). The WWTPs’ processes and sampling points are presented in Fig. 1.

Compost samples were collected from the local composting facility, where the dewatered sludge from the WWTPs is transported and combined with biowaste in a ratio of 25:75. Compost was collected by the composting facility at three different age stages: before composting (C1), after 5 days maturing outside (C2), and after 4 months maturing outside (C3).

Sediment samples were taken in July 2024 from the riverbed of Porvoonjoki River, upstream and downstream the WWTP discharge outlet as well as from the retention pond. The Porvoonjoki River is a small river, with a mean flow of 3.4 m^3^/s at the WWTP area, including the contribution from the treated effluent. Sampling points were point A retention pond, point B upstream of effluent discharge outlet, and points C, D, and E 10, 40, and 200-m downstream of the discharge outlet, respectively.

All samples were stored in the cold room (+ 4 °C) prior to the sample preparation. Prior to analysis, samples were manually mixed, and several subsamples were taken from the main lot using the grab sampling technique. The samples were then homogenized with a pestle and mortar and sieved through a 1-mm sieve.

### Sample pretreatment

#### Microwave-assisted extraction

Firstly, frozen samples were freeze-dried with an Alpha 1–4 freeze-dryer (Christ, Germany) and then weighed to the MARS6 (CEM, USA) PFA tubes in approximately 0.3 g weight. All samples were prepared in three replicates. Then, 200 ng of internal standards was added to the tubes and 20 mL of 1:1 (*v*/*v*) methanol and water (pH 2, adjusted with HCl) was added to the extraction tubes. Extraction was carried out by using an 800-W MARS6 microwave device. During the first 10 min, samples were heated to 110 °C at the power of 800 W and pressure of 30 bar, then held at this temperature for 30 min, and then slowly cooled down.

#### Solid-phase extraction

The MAE extracts were further purified with solid-phase extraction (SPE) clean-up procedure. Samples were filtered through a 1.6-μm GF/A glass fiber filter using vacuum and transferred to the 200-mL ethanol-rinsed glass bottles. Extracts were diluted with acidic water so that the total concentration of methanol was 5%. The next step was SPE performed with Oasis HBL cartridges. The SPE was done as previously optimized and described by Alitalo et al. (2022). Extracts were eluted with 3 × 3 mL of pure MeOH into glass Kimax tubes. Then, samples were evaporated under gentle nitrogen flow at a temperature of 35 °C to dryness, and 1 mL of MeOH was added to the tubes. Finally, samples were filtered through 0.2-µm PTFE membrane (Merck Millipore Ltd, Ireland) to the glass LC vials and stored in the freezer until LC–MS/MS analysis.

#### Organic matter

To measure the organic matter content of sewage sludge, sediment, and compost samples, a standard loss-of-ignition method was implemented (SFS 3008 1990). The weighted dried samples were placed in the muffle furnace and heated for 4 h at the temperature of 550 °C and cooled down to approximately 100 °C before final weighing. The organic matter content varied between 65 and 78% in sewage sludge samples, 40–52% in compost samples, and 2–8% in sediment samples (Table 2).

**Table 2:** Organic matter content in the solid samples (%, mean ± relative standard deviation, *n* = 3)

Sample type	Average organic matter content, %
Sediment A	6.5 ± 0.1
Sediment B	4.5 ± 0.0
Sediment C	8.3 ± 0.2
Sediment D	2.1 ± 0.1
Sediment E	5.3 ± 0.1
Mixed sewage sludge	78.0 ± 0.2
Fermented sewage sludge	64.9 ± 0.2
Dewatered sewage sludge	65.7 ± 0.2
Compost 0 days	52.5 ± 0.3
Composted 5 days	50.1 ± 0.4
Composted 120 days	40.4 ± 0.3

### Liquid chromatography–mass spectrometry (LC–MS/MS)

The analysis of selected pharmaceuticals was carried out by using ACQUITY™ UPLC™ liquid chromatography system (Waters Corp., Milford, MA, USA) coupled to a Micromass Quattro Premier (Waters Corp., Milford, MA, USA) triple quadrupole mass spectrometer with electrospray ionization (ESI). The chromatographic separation of compounds was done by using Waters ACQUITY™ UPLC™ HSS T3 column (2.1 × 50 mm, 1.8 µm, 100 Å). Column temperature was 30 °C and injection volume 5 µL. The mobile phase composition consisted of binary mixtures of 0.1% formic acid in ultrapure water (A) and methanol (B). Elution was done by using the following gradient: 0–4.5 min 30% B, 4.5–5.5 min 100% B, 5.5–7.0 min 100% B, and 7.1–12 min 30% B. The column was re-equilibrated for 3 min between runs and the total flow rate was 0.3 mL/min.

The mass spectrometer was operated in positive mode (ESI +) under the following conditions: source temperature 120 °C, desolvation temperature 300 °C, capillary voltage 3.0 kV, the nitrogen gas flow rates of the desolvation and nebulizing gas were 700 and 50 L/h, respectively. Argon was used as the collision gas, and the pressure of the collision chamber was maintained at 3.5 × 10^–3^ bar. Data acquisition was performed in the multiple reaction monitoring (MRM) mode. The selection of the specific MRM conditions for each of the studied pharmaceuticals was performed by injecting individual standard solutions directly into the source. Two MRM transitions per compound were acquired and the most intense one was used for quantification, while the other one was used for identification and confirmation. Cone voltage was the parameter influencing the intensity the most and it was optimized for each compound, as well as the optimum collision energy (CE). The used parameters are shown in Table 3.

**Table 3:** MS/MS parameters used for analysis of studied pharmaceuticals by MRM positive ionization mode

Compound	Precursor ion (*m/z*)	Product ion (*m/z*)	Cone voltage (CV)	Collision energy (CE)
Carbamazepine	237.0	165.0	25	40
		194.0	20	20
Diclofenac	296.0	215.0	20	15
		250.0	18	12
Letrozole	286.0	217.0	18	15
		189.9	18	32
Naproxen	231.0	170.0	30	25
		185.0	20	15
Tamoxifen	372.1	72.1	35	25
		129.0	30	25
Carbamazepine-[d_10_]	247.0	204.0	20	20
Diclofenac-[^13^C_6_]	302.0	221.0	20	15
		256.0	18	12
Letrozole-[d_4_]	290.0	221.0	18	12
		94.0	18	30
Naproxen-[d_3_]	234.0	170.0	30	30
		188.0	20	15
Tamoxifen-[d_5_]	377.2	72.1	35	25
		134.0	35	25

### Environmental risk assessment in agricultural soil

The environmental risk of carbamazepine (CBZ) originating from compost application to agricultural soils was assessed by calculating the predicted environmental concentration in soil (PEC_soil_) according to the methodology described in the European Commission Technical Guidance Document on Risk Assessment (EC-TGD 2003, EUR 20,418 EN/2) using the following equation:

1 $${{\mathrm{P}\mathrm{E}\mathrm{C}}_{\mathrm{s}\mathrm{o}\mathrm{i}\mathrm{l}}=\frac{{C}_{\mathrm{s}\mathrm{l}\mathrm{u}\mathrm{d}\mathrm{g}\mathrm{e}} \times {\mathrm{A}\mathrm{P}\mathrm{P}\mathrm{L}}_{\mathrm{s}\mathrm{l}\mathrm{u}\mathrm{d}\mathrm{g}\mathrm{e}}}{{\mathrm{D}\mathrm{E}\mathrm{P}\mathrm{T}\mathrm{H}}_{\mathrm{s}\mathrm{o}\mathrm{i}\mathrm{l}} \times {\rho}_{\mathrm{s}\mathrm{o}\mathrm{i}\mathrm{l}}}}$$

where *C*_sludge_ is the concentration of the pharmaceutical in compost in ng/g, APPL_sludge_ is the dry sludge application rate (0.6 kg/m^2^ year for agricultural soils in Finland), DEPTH_soil_ is the mixing depth of soil (0.2 m), and *ρ*_soil_ is the bulk density of wet soil (1700 kg/m^3^). These values are commonly used default values for EU environmental risk assessment and are based on typical European agricultural soil conditions.

The environmental risk was further evaluated by calculating the risk quotient (RQ) using Eq. 2:

2 $$\mathrm{R}\mathrm{Q}= \frac{\mathrm{P}\mathrm{E}\mathrm{C}}{\mathrm{P}\mathrm{N}\mathrm{E}\mathrm{C}}$$

where PNEC is the predicted no-effect concentration. A PNEC_soil_ value of 0.05 ng/g was used based on literature data (Muñoz et al. 2009; Vieno 2015). Risk levels were interpreted according to established criteria: RQ < 0.1 indicates negligible risk, 0.1–1 low risk, 1–10 moderate risk, and > 10 high risk.

### Method validation and quality assurance

For validation of the method, 200 ng of analytes was spiked into each different type of sample matrices (sludge, sediment, and compost). To calculate the absolute recovery, internal standards were added right before the analysis. To ensure the quality and reproducibility of the analysis, all samples were prepared with three replicates. Procedural blank samples were used to detect any possible contamination during the experimental procedure, and solvent blanks were used to monitor possible carryover between runs.

Internal standard calibration was used to correct for MS responses and to ensure exact quantification performance. The concentration of the internal standards was in all cases 0.04 ng/µL. The validation data are shown in Table 4. The linearity of the method was evaluated with standards at seven different concentrations, from 0 to 0.3 ng/µL. Correlation coefficients (*R*_2_) above 0.99 were obtained for all compounds over the concentration range studied. The limit of detection (LOD) and limit of quantification (LOQ) values were based on the signal-to-noise ratio (*S*/*N*). For LOD and LOQ calculation, *S*/*N* values of 3 and 10 were used, respectively. LOD and LOQ values were determined from samples and for each matrix: sludge, sediment, and compost, and the values are presented in Table 5.

**Table 4:** Quality parameters of studied compounds. M = mixed sewage sludge; F = fermented sewage sludge; A, D, and E = sampling points for sediment

Compound	Recovery rate + SD, %						Linearity (ng/µL)
	Sewage sludge		Sediment			Compost	
	M	F	A	D	E	(5 d)	
Carbamazepine	100 ± 3	108 ± 2	93 ± 5	126 ± 3	120 ± 26	96 ± 4	0.005–0.3
Diclofenac	85 ± 10	67 ± 1	75 ± 8	100 ± 6	91 ± 12	85 ± 7	0.005–0.3
Letrozole	109 ± 3	102 ± 5	88 ± 7	92 ± 3	95 ± 9	82 ± 6	0.005–0.3
Naproxen	76 ± 5	82 ± 1	88 ± 8	97 ± 8	91 ± 5	85 ± 9	0.005–0.3
Tamoxifen	107 ± 3	105 ± 1	99 ± 23	126 ± 12	101 ± 23	91 ± 4	0.005–0.3

**Table 5:** Limit of detection (LOD) and limit of quantification (LOQ) values for each studied compound in different matrices

Compound	LOD, ng/g dry weight			LOQ, ng/g dry weight		
	Sewage sludge	Sediment	Compost	Sewage sludge	Sediment	Compost
Carbamazepine	1	0.15	2	3	0.5	6.6
Diclofenac	7.6	2.2	15	25	7.3	45
Letrozole	2.3	0.3	n.d	7.6	1	n.d
Naproxen	3.4	2.5	n.d	11.2	8	n.d
Tamoxifen	1.8	0.2	0.15	6	0.66	0.5

*n.d.* not detected

## Results and discussion

### Method validation

The studied five pharmaceuticals were successfully analyzed from solid sample matrices, including sewage sludge, compost, and sediment. Developed method included efficient extraction by MAE, followed by further purification with SPE and finally UPLC-MS/MS analysis. The extraction of pharmaceuticals from solid samples is commonly done by using ultrasonic solvent extraction (USE) and pressurized liquid extraction (PLE) techniques (Jelic et al. 2009, Seira et al. 2013, Martin et al. 2012, Carballa et al. 2008a, b), whereas the MAE is seldomly used technique. However, it is well suitable for this purpose, as previously reported by Petrie et al. (2016), who detected 41 compounds, including CBZ, DCF, and NPX, from fermented sludge samples using a similar analytical method compared to our study. Vega-Morales et al. (2011) also used MAE extraction to determine estradiol-mimicking compounds from sewage sludge. According to Dorival-García et al. (2013), all three techniques are reliable, but MAE and PLE are considered the best options due to their high extraction efficiency, low solvent consumption, and simultaneous extraction of serial samples. In this study, the extraction efficiency was good, as the absolute recoveries for all compounds in all studied matrices were at the acceptable range (70–120%), except for CBZ and TAM in retention pond sediment and DCF in fermented sludge (Table 4). However, these deviations are comparatively slight and good recoveries for these pharmaceuticals in other matrices are achieved.

### Pharmaceuticals in sewage sludge

Carbamazepine and diclofenac were the most abundant compounds among the studied pharmaceuticals. CBZ was detected in all sewage sludge samples from both WWTPs (Table 6), with concentrations ranging from 29 to 92 ng/g dry weight. These results are well aligned with previous studies presenting CBZ in sewage sludge samples at concentrations varying between 10 and 258 ng/g dw (Aydin et al. 2022, Jelic et al. 2009, 2011, Miao et al. 2005, Radjenović et al. 2009). Significantly lower concentrations (< 1 ng/g) have also been reported in sewage sludge samples collected from wastewater treatment plants in southern China (Wang et al. 2025). Fermented sludge samples contained the highest concentrations of CBZ at both WWTPs; mean concentration at WWTP I was 44 ng/g dw and 92 ng/g dw at WWTP II. The measured concentration of CBZ at WWTP II is similar to previous studies by Vieno (2015) and Petrie et al. (2016), who reported CBZ in fermented sludge at a concentration of 108 and 121 ng/g dw, respectively. The difference in concentrations between the studied WWTPs can be due to WWTP II receiving higher volume of wastewater from households, whereas WWTP I receives more industrial wastewater. Seasonal variation may also partly explain the observed differences, as samples from WWTP II were collected in February, whereas samples from WWTP I were collected in July. In Finland, wastewater temperatures at WWTPs average approximately 12 ± 1 °C for about half of the year (Kruglova et al. 2014), and lower temperatures have been reported to reduce the biodegradation efficiency of pharmaceuticals during wastewater treatment processes (Vieno et al. 2005). The higher concentration of CBZ in fermented sludge compared to unfermented sludge is often reported (Dolu & Nas 2023; Yang et al. 2016; Vieno 2015). This may be due to the conjugation/deconjugation processes, as during the treatment process, conjugated forms of excreted CBZ are cleaved back to free forms by microbial activity (Radjenović et al. 2009, Miao et al. 2005). Also, the reduction of sludge mass and volume after the fermentation process leads to higher concentrations for nondegradable compounds (Vieno 2015). CBZ showed persistence across all processing stages of sewage sludge (mixed, fermented, and dewatered) indicating its resistance to degradation during wastewater treatment process. Similar results presented studies by Yang et al. (2016) and Jelic et al. (2011), who measured CBZ in primary sewage sludge in concentrations of 154 and 50 ng/g dw, respectively, with no exhibited degradation throughout sludge processing stages. Malmborg and Magnér (2015) studied the effectiveness of anaerobic digestion and six sanitization technologies to remove pharmaceuticals, including CBZ, from sewage sludge and CBZ showed persistence to most treatments except for advanced oxidation process by Fenton’s reaction.

**Table 6:** Concentrations of pharmaceuticals in the sewage sludge samples taken from WWTP I and II (ng/g dry weight, mean ± standard deviation, *n* = 3) at three processing stages: mixed (M), fermented (F), and dewatered (D) sewage sludge. Results from WWTP II are presented as two separate rounds, as during the first round no dewatered samples were analyzed

Compound	WWTP I			WWTP II (round 1)		WWTP II (round 2)		
	M	F	D	M	F	M	F	D
CBZ	29 ± 7	44 ± 4	39 ± 5.5	45 ± 2.5	89 ± 6.6	54 ± 27	92 ± 7	60 ± 0.7
DCF	140 ± 45	213 ± 69	109 ± 11	145 ± 43	70 ± 12	104 ± 7.0	91 ± 2.8	46 ± 9.5
LTZ	n.d	n.d	n.d	< LOQ (6.8)	n.d	n.d	n.d	n.d
NPX	70 ± 29	n.d	n.d	n.d	n.d	n.d	n.d	n.d
TAM	5.2 ± 2	6.3 ± 0.6	6.0 ± 0.7	45 ± 25	19 ± 3	6.6 ± 3.8	9.7 ± 4.1	6.0 ± 3.0

*n.d.* not detected, < *LOQ* below limit of quantification

Diclofenac was also detected in all sewage sludge samples, with concentrations exceeding 100 ng/g dw in every sample, except for fermented and dewatered sewage sludge from WWTP II. The highest concentration of 288 ng/g was measured in fermented sludge sample from WWTP II and the lowest (40 ng/g dw) in dewatered sludge sample from the same WWTP. These results are somewhat higher compared to previous studies, which have reported concentrations between 19 and 30 ng/g dw (Aydin et al. 2022, Petrie et al. 2016; Peysson & Vulliet 2013; Samaras et al. 2013; Yang et al. 2016), but well aligned with studies by Jelic et al. (2009), Radjenovic et al. (2009), and Vieno (2015) who reported similar concentrations of DCF varying between 28 and 200 ng/g dw. The behavior of DCF is further complicated by its ionizable nature, resulting in variable sorption depending on environmental conditions. Reported log *K*_oc_ values (2.2–3.4) suggest moderate but variable affinity to organic matter (Franco and Trapp 2008; Carballa et al. 2008b), which may explain the observed variation in concentrations during sludge processing. DCF showed slight decrease in concentrations toward the last stage of sewage sludge processing—dewatered sewage sludge—in both WWTPs, but relatively high levels of DCF in all sewage sludge samples indicate its resistance toward sludge treatment processes. This was somewhat expected, as previous studies have reported DCF to be hardly biodegradable. Peng et al. 2019 studied six different removal routes of pharmaceuticals, including DCF, in aerobic conditions, and no removal was observed. Additionally, Joss et al. (2006) and Suárez et al. (2012) concluded that this compound is hardly biodegradable. The chemical structure of DCF, especially chloro moieties, which are strong electron withdrawing functional groups, also suggests its resistance toward anaerobic digestion (Yang et al. 2016).

The other studied NSAID, naproxen, was detected only in mixed sludge samples collected from WWTP I, with a mean concentration of 70 ng/g dw (Table 6). Other studies have reported NPX in sludge samples in concentrations between 4 and 40 ng/g dw (Jelić et al. 2009, Petrie et al. 2016, Yang et al. 2016), whereas Samaras et al. (2013) reported higher concentrations in Greece (270–930 ng/g dw). NPX has relatively low sorption affinity (log *K*_oc_ 2.0–3.0, Carballa et al. 2008b), indicating high mobility in the aqueous phase and limited partitioning to sludge. However, it should be noted that log *K*_oc_ values for ionizable pharmaceuticals (e.g., DFC and NPX) are strongly influenced by environmental conditions such as pH and matrix composition and therefore should be interpreted as indicative rather than absolute descriptors of environmental behavior (Franco and Trapp 2008). Owing to their physicochemical properties, particularly the presence of carboxylic acid groups, both compounds predominantly occur in anionic form at neutral pH, which further reduces their sorption to sewage sludge (Vieno et al. 2018, Wojcieszyńska and Guzik 2020). The consumption of NPX in Finland is relatively high (Table 1), but at WWTPs, it has a fairly high removal rate of 81 ± 18%, compared to, for example, DCF which is often poorly removed (Lindqvist et al. 2005). Certain pharmaceuticals, particularly antibiotics and NSAIDs, have been reported to occur at higher concentrations during winter months, likely due to the increased incidence of seasonal illnesses and the associated higher consumption of these pharmaceuticals. For example, Aydin et al. (2022) observed elevated concentrations of NSAIDs in fermented sludge during wintertime. However, in the present study, no clear seasonal variation in NSAID concentrations was observed.

Anticancer drug LTZ was detected only in mixed sludge samples at concentrations below LOQ (6.8 ng/g dw). As LTZ is slightly lipophilic and its concentration in wastewater was low (Alitalo et al. 2022), it was hypothesized that it could be present at higher concentrations in sewage sludge. As far as we know, no previous studies on the occurrence of LTZ in sewage sludge are available, and therefore, its behavior during wastewater treatment remains largely unknown. Unfortunately, in this study, the first sludge samples were collected prior to fermentation, meaning that the sludge had already undergone clarification and aeration processes, and the presence of LTZ during these earlier treatment stages was not investigated. The overall annual consumption of LTZ in Finland is relatively low (14 kg), with a typical daily dose of 20 mg (Fimea 2024). Consequently, its concentrations in WWTP may remain below detectable levels, particularly in complex matrices such as sewage sludge. Although the overall number of studies about the occurrence of LTZ remains limited, it has been earlier detected in hospital and wastewater effluent at concentrations < 2.4 ng/L (Liu et al. 2010; Alitalo et al. 2022).

Tamoxifen was detected in sewage sludge samples from both WWTPs in concentration ranging from 5 to 45 ng/g dry weight, with WWTP II showing a bit higher level of TAM than WWTP I (Table 6). However, its detection was inconsistent, as the variation between replicates was quite high, especially in the first samples of mixed sludge from WWTP II (Table 6). In all other samples, the concentrations were similar to the study by Aydin et al. (2022), as they reported TAM in activated sludge with maximum concentration of 6.1 ng/g. Fick et al. (2011) reported concentrations of TAM in dewatered sludge samples from 6.7 to 13 ng/g dw. Interestingly, TAM did not show any signs of degradation after the sewage sludge anaerobic digestion process, which is comparable to the study by Alenzi et al. (2021). In the previous study by Alitalo et al. (2022), carried out at the same WWTPs, TAM was not detected in influent or effluent above LOQ values, apart from a few samples, where TAM was detected in a low concentration of 0.5 ng/L. Being the most lipophilic compound (log *K*_ow_ 6.3) out of all studied pharmaceuticals, TAM is expected to be bound to solids during the activated sludge process rather than be transported to receiving waters with wastewater effluent. In the literature, however, TAM is frequently detected in wastewater and aquatic environments. Previously reported concentrations varied between 10 and 369 ng/L in wastewater effluents (Ashton et al. 2004; Roberts and Thomas 2006; Ferrando-Climent et al. 2014), and the highest reported concentration in surface water is 212 ng/L (Roberts and Thomas 2006). Despite its presence in aquatic environments, literature on TAM occurrence in sewage sludge remains extremely scarce. In addition to the studies by Aydin et al. (2022) and Fick et al. (2011), only a few studies investigated TAM in sewage sludge samples but did not detect it above LOQ values (Santana-Viera et al. 2020; Petrie et al. 2016; Peysson and Vulliet 2013). To the best of our knowledge, this is the first determination of TAM in Finnish sewage sludge.

### Pharmaceuticals in compost

Carbamazepine was detected in all compost samples throughout the maturing stages with concentrations ranging from 5 to 15 ng/g dw, suggesting that even during the composting process, CBZ is not completely degraded (Table 7). Although the concentration of CBZ showed a slight decrease during composting, it was still detected after 120 days above the concentration of 5 ng/g dw. The literature of pharmaceuticals in compost matrix is scarce, but Bastos et al. (2020) reported CBZ in composted sludge at a mean concentration of 112 ng/g dw. Vieno (2015) reported CBZ in compost samples at a concentration of 25 ng/g dw. The consistent occurrence in sewage sludge in both WWTPs as well as its detection in compost samples confirms CBZ’s persistence and resistance to degradation.

**Table 7:** Concentrations of CBZ, DCF, LTZ, NPX, and TAM in compost samples (ng/g dry weight, mean ± relative standard deviation, *n* = 3) taken from three different age stages: at the start of the composting (C1), 5 days curing outside (C2), and 4 months curing outside (C3)

Compound	14.9.2023			27.9.2023		
	C1	C2	C3	C1	C2	C3
CBZ	15 ± 1.2	15 ± 1.3	9.3 ± 1.0	8.4 ± 0.2	10.0 ± 0.9	5.2 ± 1.0
DCF	< LOQ (39 ± 16)	n.d	< LOQ (23 ± 4)	< LOQ (34 ± 13)	< LOQ (31^*^)	< LOQ (41 ± 8)
LTZ	n.d	n.d	n.d	n.d	n.d	n.d
NPX	n.d	n.d	n.d	n.d	n.d	n.d
TAM	n.d	n.d	n.d	n.d	n.d	n.d

^*^Only one replicate

Diclofenac was also detected in all compost samples, with one exception (14.9.2023, 5 days, Table 7). The LOQ for DCF in compost matrix was high (45 ng/g dw), and therefore, the average detected concentrations (shown in parentheses in Table 7) stay below LOQ. The highest concentration (60 ng/g dw) was detected in 0-day sample during first sampling round. The detected concentrations are aligned with concentrations from previous studies, as Styszko et al. (2022) detected DCF in fertilizers containing sewage sludge at concentrations between 36 and 52 ng/g dw and Bastos et al. (2020) measured DCF in composted sludge at mean concentration of 37 ng/g dw. Although the concentrations in this study were below LOQ, it was detected in most of the samples, indicating its persistence. Due to the dilution with biowaste, the compost contains only 25% of dewatered sludge, which most likely lowered the concentrations to levels below LOQ.

In compost samples, TAM was not detected at all, suggesting either significant degradation during composting process or, more likely, dilution effect due to addition of biowaste in composting mix. As the occurrence of TAM in sewage sludge was generally below 10 ng/g, dilution with biowaste in 1:4 probably explains its absence in compost samples. However, it should be noted that in some countries sewage sludge is not subjected to further treatment processes such as composting, which may result in the release of TAM into agricultural soils. The potential risks of TAM and LTZ to aquatic organisms have been studied, showing effects on reproductive capacity, vitellogenin levels, and transgenerational impacts in offspring (Liao et al. 2014; Sun et al. 2007a, b). In addition, both compounds are considered potentially carcinogenic, while their adverse effects on soil organisms remain largely unknown. LTZ and NPX were not detected in any compost samples, which was expected according to their results in sludge samples.

The CBZ concentrations determined in compost can be used to estimate the potential entry of the pharmaceutical into agricultural soils by calculating the predicted environmental concentration (PEC_soil_). This was calculated according to the methodology described in the European Commission’s Technical Guidance Document on Risk Assessment (EC-TGD 2003, EUR 20,418 EN/2) using the Eq. 1 (see “Environmental risk assessment in agricultural soil”). The predicted environmental concentration of CBZ in soil following the application of compost containing 9 ng/g of the compound was calculated to be approximately 16 ng/kg reflecting strong dilution in the soil matrix. If sewage sludge was applied to agricultural soil without prior composting, the predicted environmental concentration in soil (PEC_soil_) would be approximately 106 ng/kg, using the concentration of dewatered sludge from WWTP II. This highlights the importance of further treatment of dewatered sludge to reduce the potential environmental exposure of contaminants.

Vieno et al. (2018) published a report on the potential risks posed by contaminants present in fertilizers containing sewage sludge. According to their results, CBZ is fairly mobile in soil, and they classified CBZ as a persistent compound in soil. Styszko et al. (2022) also reported high mobility of CBZ in their study of fertilizer leachates. In this study, the environmental risk of CBZ was estimated by calculating risk quotient (RQ). If the RQ value is below 0.1, no adverse effect is expected, but values between 0.1 and 1 suggest low risk but the potentiality of adverse effects should be taken into consideration. Higher values between 1 and 10 indicate some adverse effect or moderate risk, and RQ values above 10, a high risk is anticipated. Vieno et al. (2018) calculated RQ values for CBZ using PNEC_soil_ value and sewage sludge concentrations from literature (Muñoz et al. 2009; Vieno 2015), and their results suggest that CBZ belongs to a group of contaminants that threatens soil organisms (RQ 4). Using the same PNEC_soil_ value (0.05 ng/g) and PEC_soil_ value obtained in this study, the calculated RQ is 0.2. It should be noted that this RQ value considers only the time of fertilizer application, and it does not include cumulative applications. Fertilizers and soil amendments are often spread several times on the same spot, and as some compounds (e.g., CBZ) are persistent, their concentrations may increase in the soil, leading to bioaccumulation in soil organisms and food chains (Vieno et al. 2018). A previous study has demonstrated that plants may uptake pharmaceuticals present in soil, and, for example, carbamazepine tends to accumulate in plant leaves (Wu et al. 2013). Knight et al. (2017) further showed that carbamazepine can also be translocated to zucchini fruits and reported that high carbamazepine concentrations (> 14 mg/kg) negatively affected fruit production.

### Pharmaceuticals in sediment samples

Majority of the studied pharmaceuticals were found in most of the river sediment sampling points at low concentrations (Table 8). The highest concentration of CBZ was detected in the retention pond (3.5 ng/g dry weight), followed by sediments C, D, and E. These findings indicate its distribution following the river flow dynamics, as the concentrations decreased with increasing distance from the effluent discharge outlet due to dilution and dispersion. Similarly, DCF was detected in the highest concentration in the retention pond sediment (11.0 ng/g dry weight), followed by sediment D (4.6 ng/g dry weight) and sediment C (3.0 ng/g dry weight). It was not detected in sediment E. Despite its absence in sewage sludge and compost samples, NPX was detected in sediment samples, showing the similar distribution pattern as CBZ and DCF; LTZ and TAM were not detected in any of the sediment samples. A previous study by Azuma (2018) reported low concentrations of TAM in surface water and sediment, with concentration ranges of 0–5 ng/L and 0.04–0.25 ng/g, respectively.

**Table 8:** Concentrations of CBZ, DCF, LTZ, NPX, and TAM (ng/g dry weight, mean ± standard deviation, *n* = 3) in the sediment samples from Porvoonjoki River and Nikula retention pond at sampling point A—Nikula retention pond, point B—upstream from effluent discharge pipe, point C—10-m downstream from effluent discharge pipe, point D—40-m downstream from effluent discharge pipe, and point E—200-m downstream of discharge pipe

Compound	Concentration, ng/g dry weight (average + SD)				
	A	B	C	D	E
CBZ	3.5 ± 0.6	n.d	2.1 ± 0.3	1.9 ± 0.3	< LOQ (0.3)
DCF	11.0 ± 2.5	n.d	3.0 ± 0.2	4.6 ± 1.2	n.d
LTZ	n.d	n.d	n.d	n.d	n.d
NPX	4.5 ± 2.2	n.d	4.0 ± 0.3	4.8 ± 2.8	n.d
TAM	n.d	n.d	n.d	n.d	n.d
OM %	6.5	4.5	8.3	2.1	5.3

*n.d.* not detected, < *LOQ* below limit of quantification

While literature on LTZ and TAM occurrence in sediments remains limited, CBZ has been frequently detected rather in WWTP effluent than in sediment (Yang et al. 2011). CBZ is one of the least lipophilic chemicals among the studied pharmaceuticals with a log *K*_ow_ of 2.45; therefore, it tends to be mostly found in liquid phases rather than solids. In the Finnish aquatic environment, CBZ and DCF are previously detected in Lake Päijänne sediment at average concentrations below 1 and 3 ng/g (Lindholm-Lehto et al. 2015). Globally, CBZ is frequently detected in concentration ranges from high ng/L to low µg/L in both effluent and surface water (Nieto et al. 2017; Hai et al. 2018). However, concentrations in sediment of these compounds were reported less often. Compared to this study, CBZ was found at slightly higher concentrations (0.6–24 ng/g) in Lake Victoria sediment, which is rich in organic matter (Nantaba et al. 2024), whereas Wang et al. (2025) detected CBZ only below LOQ levels. Similarly, NPX has been detected in river sediment in ranges of 7–57 ng/g concentration (Wojcieszyńska and Guzik 2020).

Overall, the distribution pattern of CBZ, DCF, and NPX is quite similar, indicating similar behavior of these chemicals in the aquatic environment. CBZ is well known for its persistence in the environment, and therefore, it has been proposed as a marker for anthropogenic contamination (Clara et al. 2004). DCF (log *K*_oc_ 2.2–3.4) and NPX (log *K*_oc_ 2.0–3.0) typically occur in the environment in their negatively charged forms, which reduces their affinity for sediment binding and favors their persistence in the aqueous phase. Previous studies have shown that both compounds undergo degradation under sunlight (Packer et al. 2003); however, due to their continuous emissions, they may remain present in the environment at measurable concentrations. TAM is highly lipophilic, and it has very low water solubility, indicating its tendency to accumulate in solid matrices. In this study, it was found in sewage sludge, but due to its low concentrations in effluents, its absence in sediment is not surprising. Additionally, the low organic matter content in sediment diminishes the potential accumulation of lipophilic compounds. Sediment samples were collected only once, in July, and therefore, seasonal comparisons could not be performed. Seasonal variation might be particularly evident in the concentrations of photodegradable compounds, such as DCF and NPX, as sunlight availability in Finland is limited during wintertime. In addition, as previously mentioned, biodegradation processes are generally slower under cold conditions (Meierjohann et al. 2016; Vieno et al. 2005).

## Conclusions

In this study, the occurrence of the five selected pharmaceuticals was studied in sewage sludge, compost, and sediment matrices to assess their distribution in the solid phases of wastewater treatment and the environment using the MAE extraction method coupled with SPE and LC–MS/MS. The findings indicate that the developed method is suitable for the extraction of investigated compounds from solid matrices. Good recoveries were achieved for pharmaceuticals in sewage sludge and compost as well as in the sediment samples. However, data variability highlights the need for careful sample homogenization and increased number of sample replicates in future research. The results revealed a distinct pattern in occurrence and distribution of the selected pharmaceuticals. CBZ and DCF were the most abundant compounds found in sewage sludge, compost, and sediment samples. CBZ showed persistence across all processing stages of sewage sludge (mixed, fermented, and dewatered) indicating its resistance to degradation during wastewater treatment process. These results reflect the persistence and widespread use of CBZ and DCF. In contrast, TAM was found at low levels in sewage sludge samples, while LTZ was detected only below LOQ in a few sewage sludge samples. This finding aligns with our previous study conducted on liquid samples from the same WWTPs, where LTZ and TAM were detected in quite low concentrations, suggesting that overall contamination by these pharmaceuticals in Finnish aquatic and terrestrial environments is minimal. NPX, despite its absence in sewage sludge and compost samples, was found in sediment, indicating its potential persistence in aquatic environments. CBZ was the only compound found in compost samples at concentrations between 5 and 15 ng/g dw, indicating its persistence through various treatment processes. The estimated RQ value of 0.2 for CBZ indicates a low environmental risk. However, this assessment may underestimate the actual risk, as it does not account for repeated applications of sludge-derived fertilizers or the combined effects of multiple contaminants, which may collectively pose adverse effects on soil organisms.

Although the EU legislation does not yet mandate the removal of pharmaceuticals from wastewaters, the regulation is expected to change with the updated Urban Wastewater Treatment Directive (2024/3019/EC). This Directive introduces future deadlines for implementing tertiary treatment processes in urban WWTPs. Furthermore, proposed revisions to Directive 2008/105/EC are expected to designate several pharmaceuticals, including CBZ and DCF, as priority substances. These legislative advancements show a promise for mitigating the continuous discharge of pharmaceuticals into aquatic environments. The results of this study indicate that some of the investigated compounds readily bind to sewage sludge, suggesting their potential presence in the final composted product and, consequently, in soils. Further research is needed to improve understanding of the degradation behavior of pharmaceuticals during sludge and compost treatment processes and to evaluate their environmental impacts, even at low concentrations. As the present study focused solely on parent compounds, future studies should also investigate pharmaceutical metabolites, which may exhibit greater persistence or toxicity than the original compounds (Ferrando-Climent et al. 2017).

## Data Availability

All data generated or analyzed during this study are included in this published article.
